# Assessment of Tropism and Effectiveness of New Primate-Derived Hybrid Recombinant AAV Serotypes in the Mouse and Primate Retina

**DOI:** 10.1371/journal.pone.0060361

**Published:** 2013-04-09

**Authors:** Peter Charbel Issa, Samantha R. De Silva, Daniel M. Lipinski, Mandeep S. Singh, Alexandre Mouravlev, Qisheng You, Alun R. Barnard, Mark W. Hankins, Matthew J. During, Robert E. MacLaren

**Affiliations:** 1 Nuffield Laboratory of Ophthalmology, Nuffield Department of Clinical Neurosciences, University of Oxford, Oxford, United Kingdom; 2 Department of Molecular Medicine and Pathology, The University of Auckland, Auckland, New Zealand; 3 Department of Molecular Virology, Immunology and Medical Genetics, Ohio State University, Columbus, Ohio, United States of America; 4 Moorfields Eye Hospital Foundation Trust and UCL Institute of Ophthalmology National Institute for Health Research Biomedical Research Centre, London, United Kingdom; 5 Oxford Eye Hospital, Oxford University Hospitals NHS Trust and National Institute for Health Research Biomedical Research Centre, Oxford, United Kingdom; Radboud University Nijmegen Medical Centre, The Netherlands

## Abstract

Adeno-associated viral vectors (AAV) have been shown to be safe in the treatment of retinal degenerations in clinical trials. Thus, improving the efficiency of viral gene delivery has become increasingly important to increase the success of clinical trials. In this study, structural domains of different rAAV serotypes isolated from primate brain were combined to create novel hybrid recombinant AAV serotypes, rAAV2/rec2 and rAAV2/rec3. The efficacy of these novel serotypes were assessed in wild type mice and in two models of retinal degeneration (the *Abca4^−/−^* mouse which is a model for Stargardt disease and in the *Pde6b^rd1/rd1^* mouse) in vivo, in primate tissue ex-vivo, and in the human-derived SH-SY5Y cell line, using an identical AAV2 expression cassette. We show that these novel hybrid serotypes can transduce retinal tissue in mice and primates efficiently, although no more than AAV2/2 and rAAV2/5 serotypes. Transduction efficiency appeared lower in the *Abca4^−/−^* mouse compared to wild type with all vectors tested, suggesting an effect of specific retinal diseases on the efficiency of gene delivery. Shuffling of AAV capsid domains may have clinical applications for patients who develop T-cell immune responses following AAV gene therapy, as specific peptide antigen sequences could be substituted using this technique prior to vector re-treatments.

## Introduction

Inherited retinal dystrophies such as retinitis pigmentosa (RP) have until recently been untreatable. Recent developments in retinal gene therapy have led to the successful treatment of patients with Leber’s congenital amaurosis (LCA) due to mutations in *RPE65* [1], [2], [3], [4], [5], [6], [7]. Although the studies were designed to investigate safety, lasting functional improvements in vision have been detected in some patients [1], [6]. Importantly, these proof-of-principle studies have not suggested any relevant safety concerns with adeno-associated viral (AAV) vectors. Indeed, an AAV clinical trial has recently been started to treat choroideremia, which involves vector delivery to the fovea whilst it is still healthy, in order to prevent degeneration (NCT01461213).

While current retinal gene therapy trials have shown AAV vectors to be safe, there remain ongoing efforts to improve efficiency of gene delivery and to restrict vector tropism to specific retinal cell populations by modifying the capsid protein and/or the expression cassette. In the former case it has recently been shown that recombinant packaging of the AAV2 expression cassette into an AAV8 capsid (rAAV2/8) significantly enhanced transduction of primate photoreceptors when assessed by expression of green fluorescent protein (GFP) [8]. Furthermore the AAV5 capsid is known to transduce primate foveal cones effectively, as evidenced by restoration of trichromatic color vision in the macaque following treatment of dichromats with rAAV2/5 vector encoding the long (L) opsin gene [9].

The use of these recombinant vectors, in which a transgene cassette flanked by AAV2 inverted terminal repeats (ITR) is packaged into the capsid from another AAV serotype, is becoming established in retinal gene therapy. What has yet to be explored in detail, however, is how AAV vectors might behave if the capsid protein itself were a hybrid of several different serotypes, possibly combining beneficial effects of each for targeting a specific class of retinal cell. Furthermore, the ability to shuffle or remove antigenic capsid sequences that may induce an immune response, would be of benefit in systemic gene therapy where high vector doses are used, or in treating the second eye in ocular gene therapy.

Since the discovery of AAV8 through PCR-based screening of human and primate tissues, several further AAV serotypes have been identified. [10] Analysis of the infectivity of these new serotypes in the mouse brain identified three serotypes (cy5, rh20 and rh39) that had similar tropism to, but greater infectivity than, rAAV2/8 [11].

The purpose of this pilot study was therefore to explore the possibility that structural domains might be exchanged between capsids of different serotypes in order to confer greater infectivity or to alter cellular tropism. Fragments of capsid sequences from the newly discovered primate serotypes were combined with AAV8 to create vectors with hybrid recombinant capsids (rAAV2/Rec2 and rAAV2/Rec3), and their tropism in mouse and primate retina was compared to that of rAAV2/2 and rAAV2/5. In addition to subretinal and intravitreal delivery in wild type (WT) C57BL/6 mice, subretinal injections were performed in the *Abca4^−/−^* mouse (a model for Stargardt disease) and in the *Pde6b^rd1/rd1^* mouse. The latter has no photoreceptors in advanced stages of degeneration and therefore presents a model for optobionic strategies to restore vision. In these models, high levels of AAV transduction would most likely be required to aid recombination of two halves of a split transgene (*Abca4*), or to target bipolar cells which are poorly transduced by most AAV serotypes. We also assessed tropism in the primate retina in an *in vitro* system which facilitates long term preservation of photoreceptors [12]. Finally, we tested the ability of the same viral vectors to transduce the human derived neuroblastoma cell line SH-SY5Y *in vitro*, under culture conditions that induce neuron-specific differentiation.

## Results

### *In vivo* Measurement of Fluorescence Intensity

**Figure 1:**
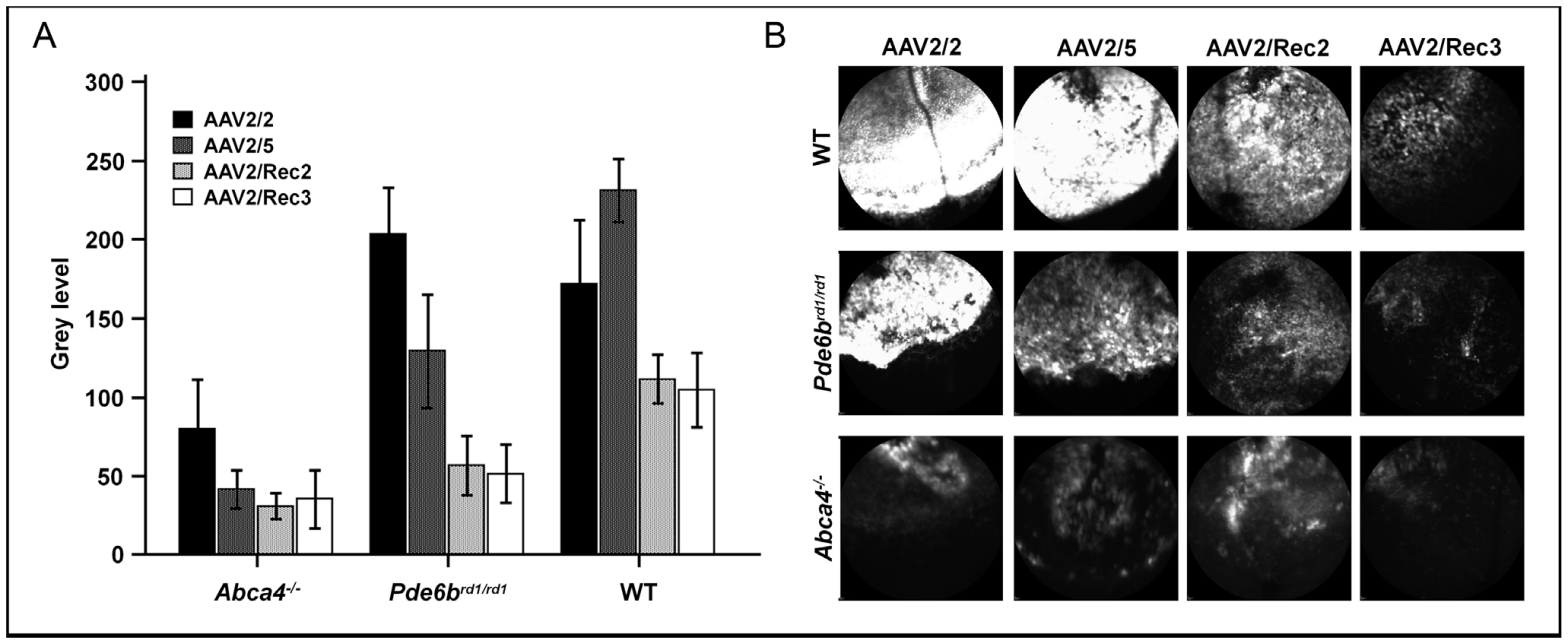
(a) Analysis of *in vivo* measurement of fluorescence intensity 3 weeks after subretinal injection of different hybrid recombinant AAV serotypes expressing green fluorescent protein (mean±SEM). Besides wild type (WT) mice, a strain with totally degenerate retina (Pde6b^rd1/rd1^ mouse) as well as a strain with strong lipofuscin accumulation in the retinal pigment epithelium (*Abca4^−/−^* mouse) were used. 4 to 8 eyes were analysed per group. (**b**) Representative topographical confocal scanning laser ophthalmoscopy (cSLO) 488 nm-fluorescence images showing the range of fluorescence intensities that was found in the different groups.

Three weeks after subretinal injection, GFP fluorescence was recorded within the area of subretinal vector injection using *in vivo* confocal scanning laser ophthalmoscopy (cSLO) imaging. All vectors tested were capable of driving GFP expression in all of the mouse strains used. GFP fluorescence did not extend noticeably outside the area of the bleb into which the vector was injected. However, clear differences in GFP fluorescence intensity were observed between mouse strains and rAAV serotypes (2-way ANOVA; p<0.001 for both strain and serotype; Figure 1). An interaction was detected between strain and rAAV serotype (p = 0.013), suggesting differing effects of the rAAV serotypes depending on retinal pathology.

Post hoc analysis showed that retinal GFP expression after viral transduction was lower in *Pde6b^rd1/rd1^* mice than in WT controls (p = 0.02), and lowest in the *Abca4^−/−^* mice (p<0.001 when compared to both other strains). Furthermore, GFP fluorescence intensity was higher with the use of rAAV2/2 than with rAAV2/Rec2 and rAAV2/Rec3 (p<0.001 for both comparisons). Fluorescence was higher with rAAV2/5 than rAAV2/Rec3 (p = 0.04) and showed a trend of superiority over AAV2/Rec2 (p = 0.07). GFP fluorescence intensities after transduction with rAAV2/2 and rAAV2/5 were similar, as was the case when comparing rAAV2/Rec2 and rAAV2/Rec3.

### Tropism of rAAV2/Rec2 and rAAV2/Rec3 in WT Mice and Disease Models

Histology was analyzed to assess retinal cell tropism of the different rAAV serotypes. Retinal cell types were identified using immunocytochemistry and by morphology.

**Figure 2:**
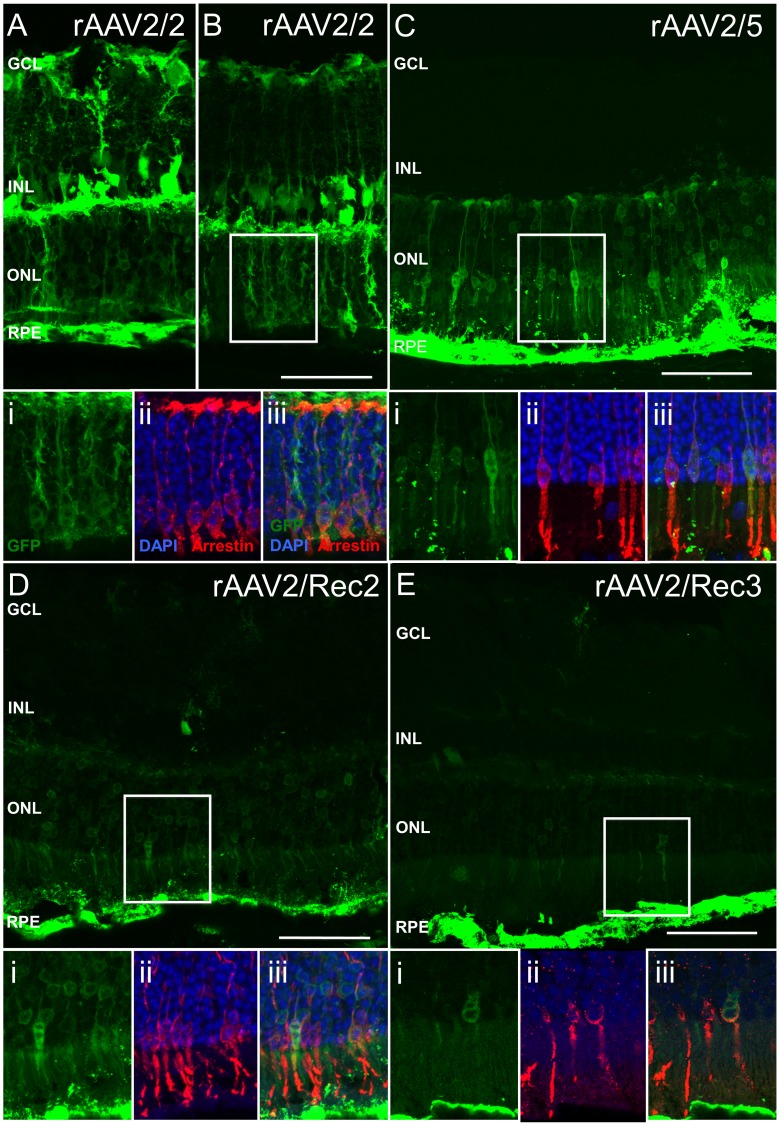
Green fluorescent protein (GFP) fluorescence patterns of different recombinant AAV serotypes in wild type C57BL/6 mice following subretinal injection. The main images A–E are confocal stacks illustrating overall GFP expression patterns. Figure A illustrates transduction of the retinal pigment epithelium with rAAV2/2, whereas figure B demonstrates cone transduction. The boxed regions in images B–E are enlarged and shown as confocal slices in panels i-iii below for each serotype. GFP signal (green) and nuclear labeling with DAPI (blue) overlaid with immunostaining using cone arrestin (red) illustrate cone transduction using the different recombinant AAV serotypes. GCL = ganglion cell layer, INL = inner nuclear layer, ONL = outer nuclear layer, RPE = retinal pigment epithelium. Scale bar 50 µm.

Following subretinal injection in WT mice, RPE cells were transduced by all serotypes tested (Figure 2). Photoreceptors were also transduced by all serotypes but GFP fluorescence intensity in this layer was higher using rAAV2/2 and rAAV2/5 than with rAAV2/Rec2 and rAAV2/Rec3. Sparse Müller cell transduction was seen with rAAV2/5 and rAAV2/Rec2, however good horizontal, Müller and ganglion cell transduction was noted with rAAV2/2 (Table 1 and Figure 2).

**Table 1:** Summary of transduction efficiency in various mouse models (subretinal delivery) and ex-vivo primate retina of the tested recombinant AAV vectors rAAV2/Rec2 and rAAV2/Rec3 in comparison to rAAV2/2 and rAAV2/5.

	RPE cells	Photoreceptors	Bipolar cells	Horizontal cells	Müller cells	RGCs
**Wild type C57/bl6**						
AAV2/2	++	++	−	++	+++	+
AAV2/5	+++	+++	−	−	+	−
AAV2/Rec2	++	++	−	−	+/−	−
AAV2/Rec3	++	+	−	−	+/−	−
***Abca4^−/−^***						
AAV2/2	++	+	−	++	++	+
AAV2/5	+	+	−	−	+/−	−
AAV2/Rec2	+	+	−	−	+/−	−
AAV2/Rec3	+/−	+/−	−	−	+/−	−
***Pde6b^rd1/rd1^***						
AAV2/2	+++	n.a.	−	+	+++	+
AAV2/5	+++	n.a.	−	+	+++	+
AAV2/Rec2	+++	n.a.	−	+	+++	+
AAV2/Rec3	+	n.a.	−	+	+	+
**Primate retina (ex vivo)**						
AAV2/Rec2	n.a.	−	−	−	−	+
AAV2/Rec3	n.a.	+	−	−	+	+++

n.a. = not applicable. RPE = retinal pigment epithelium. RGCs = retinal ganglion cells.

Following intravitreal injection, ganglion cell and Müller cell transduction was demonstrated with rAAV2/2, but there was no significant retinal transduction with rAAV2/5, rAAV2/Rec2 or rAAV2/Rec3 (data not shown).

**Figure 3:**
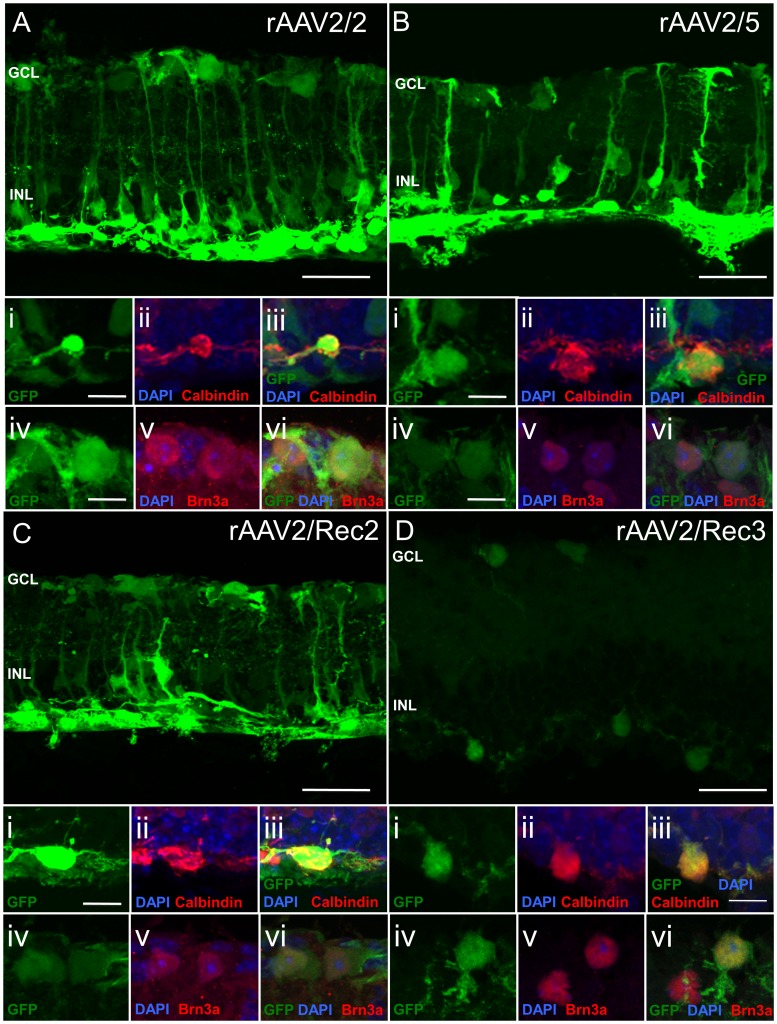
Green fluorescent protein (GFP) fluorescence patterns of different recombinant AAV serotypes in the degenerate retina of *Pde6b^rd1/rd1^* mice following subretinal injection. The main images a–d are confocal stacks illustrating overall GFP expression patterns. For each serotype, images i–iii are confocal slices showing GFP expression (green), nuclear labeling (blue) and immunostaining with calbindin identifying horizontal cells (red). Colocalisation of signals indicates horizontal cell transduction. Images iv–vi show colocalisation of GFP (green) in ganglion cells identified by Brn-3a immunostaining (red) demonstrating ganglion cell transduction. GCL = ganglion cell layer, INL = inner nuclear layer, RPE = retinal pigment epithelium. Scale bar: 30 µm for images a–d, 10 µm for images i–vi.

In the degenerate retina of *Pde6b^rd1/rd1^* mice, all serotypes transduced RPE, horizontal cells, Müller cells and ganglion cells (Figure 3). GFP fluorescence was less overall when using rAAV2/Rec3 than other serotypes and especially so for RPE and Müller cells. No bipolar cell transduction was seen with any serotype.

**Figure 4:**
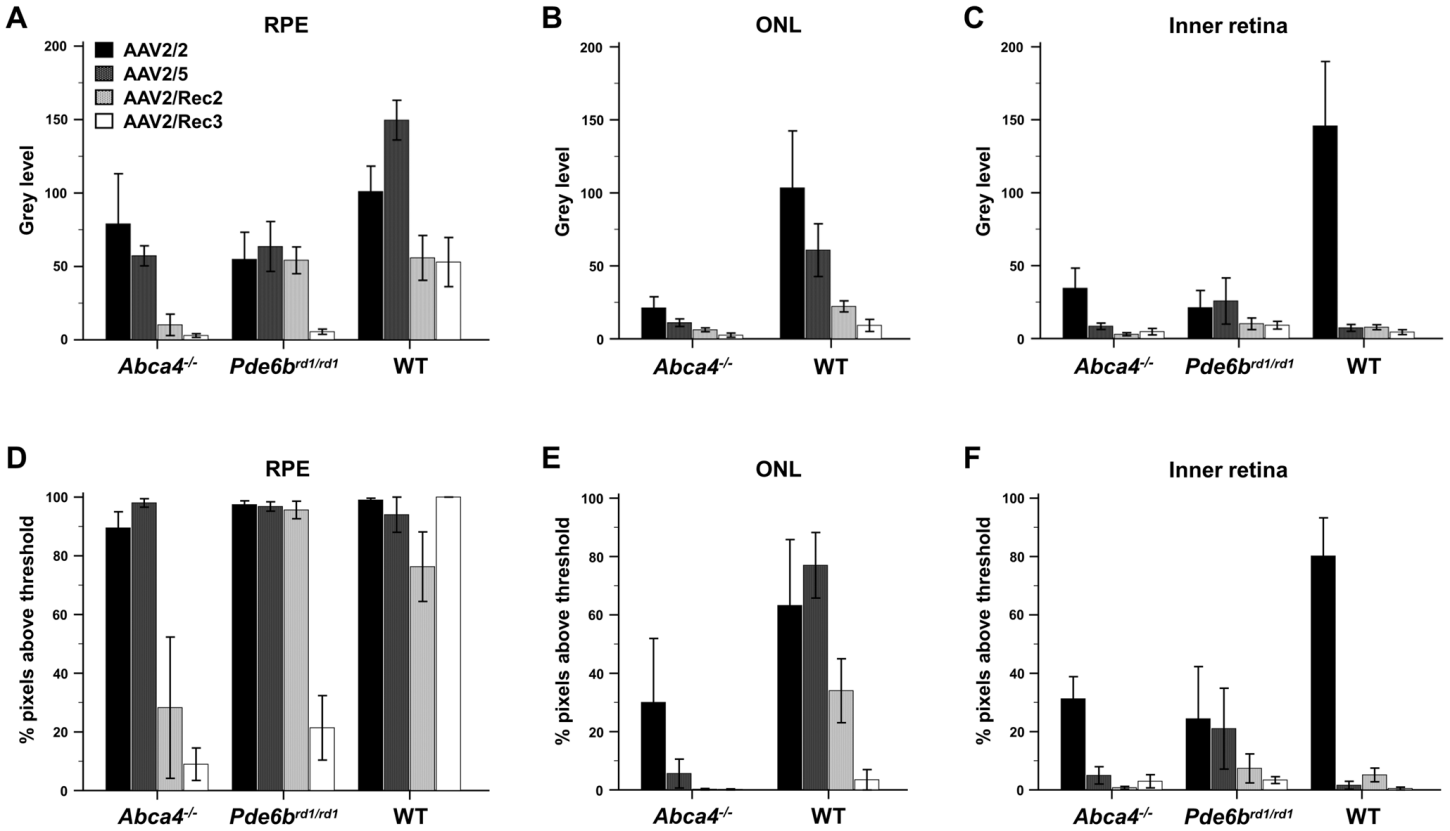
Quantitative analysis of green fluorescent protein (GFP) fluorescence intensity on histological sections in eyes that underwent subretinal injection of different recombinant AAV serotypes expressing GFP (mean±SEM). Two mouse models for retinal degeneration (*Abca4^−/−^* and *Pde6b^rd1/rd1^* mice) are compared with wild type (WT) mice. Grey level analysis (upper row) represented an estimate for the level of transgene expression within transduced cells, whereas the percentage (%) of pixels above threshold (bottom row) represented an estimate of viral efficacy for cell transduction. 4 to 8 eyes were analysed per group. ONL = outer nuclear layer, RPE = retinal pigment epithelium.

The cell types transduced by all rAAV serotypes in *Abca4^−/−^* mice were similar to those in wild type mice, but transduction was generally less effective in this model (Figure 4).

### Histological Analysis of Fluorescence Intensity

*In vivo* fluorescence intensity measurements using topographic cSLO images reflect the combined fluorescence intensity at each location arising from all retinal layers. In order to quantify the transduction efficiency within retinal layers, fluorescence intensity was measured *post-mortem* on fixed histological sections (Figure 4) of eyes with subretinal vector delivery. The level of transgene (GFP) expression within the retinal pigment epithelium (RPE) layer, photoreceptor layer, and inner retina (including the outer plexiform layer and inner nuclear layer) was quantified by grey level analysis separately in each layer. In addition, the percentage of pixels above threshold was calculated from histogram analysis, to estimate the efficiency of viral transduction.

There were significant differences in GFP fluorescence intensity between mouse strains and rAAV serotypes on histological analysis, correlating with the cSLO *in vivo* data. Grey level analysis for the RPE layer (Figure 4A) again revealed an overall effect of viral capsid serotype on the resultant GFP fluorescence (p<0.001). Post hoc analysis showed that greater RPE fluorescence was achieved with rAAV2/5 than with rAAV2/Rec2 (p = 0.02) and rAAV2/Rec3 (p<0.001). RPE fluorescence was more intense with rAAV2/2 than with rAAV2/Rec3 (p<0.001) and showed a trend of superiority compared to rAAV2/Rec2 (p = 0.06). There was no significant difference in RPE fluorescence intensity when comparing rAAV2/Rec2 and rAAV2/Rec3. Fluorescence levels were similar in *Abca4^−/−^* and *Pde6b^rd1/rd1^* mice, but both were significantly lower than WT (p<0.001).

Analysing the fraction of pixels above threshold demonstrated that among the retinal laminae analyzed, the RPE layer was the most efficiently transduced (Figure 4D). We found that rAAV2/2 and rAAV2/5 were each more effective at RPE transduction than either of the hybrid recombinant serotypes (Figure 3D). An interaction between strain and rAAV serotype (p<0.001) was seen due to the lower efficiency of rAAV2/Rec2 and rAAV2/Rec3 in transducing RPE cells in *Abca4^−/−^* mice (Figure 4D).

Analysis of transduction in the outer nuclear layer (ONL; Figure 4B,E) was performed only in *Abca4^−/−^* and WT mice as the ONL was almost completely degenerated in *Pde6b^rd1/rd1^* mice. There were significant differences in ONL transduction between strains and rAAV serotypes (2-way ANOVA; both, p<0.01). The ONL of *Abca4^−/−^* mice revealed a lower grey level (p<0.01; Figure 4B) a lower percentage of pixels above threshold intensity (p<0.001; Figure 4E) than in WT for all vectors. Comparing ONL grey level when using different vectors, rAAV2/2 was more effective than both rAAV2/Rec2 (p = 0.01) and rAAV2/Rec3 (p<0.01; Figure 4B). This could be due to the high proportion of Müller cells transduced with rAAV2/2, whose processes traverse the ONL. The percentage of ONL pixels above threshold was significantly lower when using rAAV2/Rec3 than rAAV2/2 (p<0.01) and a trend of lower efficiency than rAAV2/5 (p = 0.06), suggesting lower transduction efficiency of rAAV2/Rec3 in photoreceptors.

Analysing the effects of subretinal vector delivery within the inner retina (including the inner nuclear and inner plexiform layers), the grey level was significantly higher when using rAAV2/2 compared to all other viruses tested (all, p<0.001; Figure 4C,F). A significant difference was detected in the grey level of wild type mice in comparison with *Abca4^−/−^* and *Pde6b^rd1/rd1^* mice (p<0.05).

### Tropism of rAAV2/Rec2 and rAAV2/Rec3 Serotypes in *ex vivo* Primate Retinal Culture

**Figure 5:**
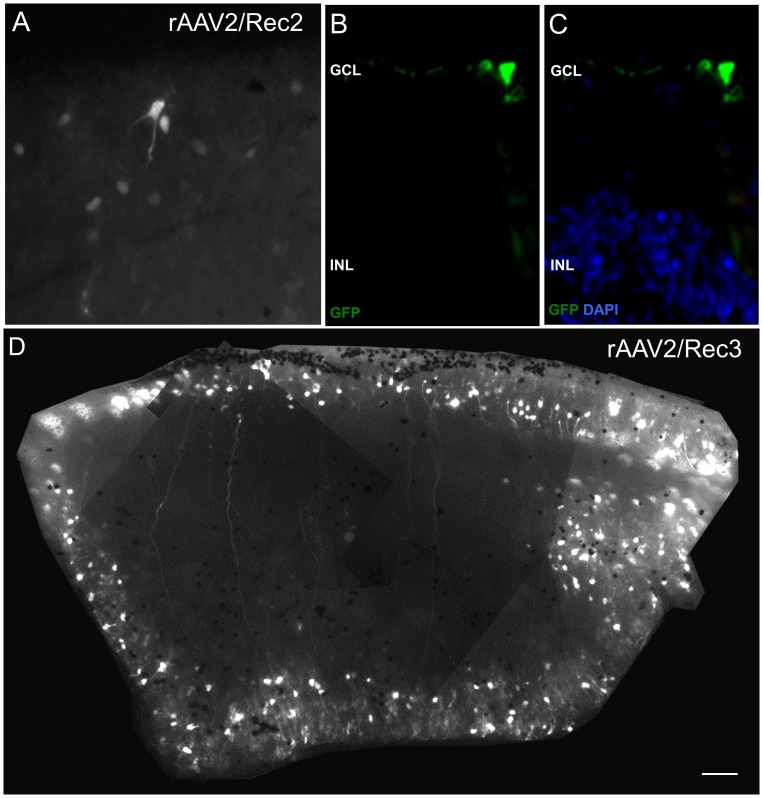
Green fluorescent protein (GFP) fluorescence following *ex vivo* administration of hybrid recombinant AAV vectors. GFP expression in macaque explants following rAAV2/Rec2 transduction (A) and histology of cross-sectional specimens showing expression of GFP (green) in the ganglion cell layer (B,C). Image D shows GFP expression in macaque explants following rAAV2/Rec3 transduction. GCL = ganglion cell layer; INL = inner nuclear layer.

*Ex vivo* culture of macaque retinal explants was used as an alternative to *in vivo* administration to assess the tropism of novel rAAV2/Rec2 and rAAV2/Rec3 serotypes in the primate retina. GFP expression was observed in explants cultured with rAAV2/Rec2 and rAAV2/Rec3 by day 3 (data not shown). *Ex vivo* fluorescence imaging of rAAV2/Rec2-cultured explants revealed limited transduction of cells on the inner retinal aspect (Figure 5A); analysis of histological sections showed these cells to be localized in the ganglion cell layer (Figure 5B–C).

Imaging of rAAV2/Rec3-cultured explants showed widespread transduction of cells on the inner retinal aspect with processes extending the width of the tissue (Figure 5D). Transduced cells were predominantly observed at the edge of the explanted retinal tissue, indicating that cellular access is restricted in the tissue’s center. This is potentially due to the presence of an intact inner limiting membrane, which is disrupted by dissection at the periphery of the tissue. The processes of transduced cells were parallel, which is suggestive of ganglion cell axons (Figure 5D). Histological analysis confirmed that rAAV2/Rec3 primarily transduced cells located in the ganglion cell layer, in addition to Müller glia (data not shown). No significant transduction of cells was observed in the ONL. Transduced cell types were determined by morphology and location within the retina.

### Measurement of GFP Fluorescence Intensity in SH-SY5Y Cells *in vitro*

**Figure 6:**
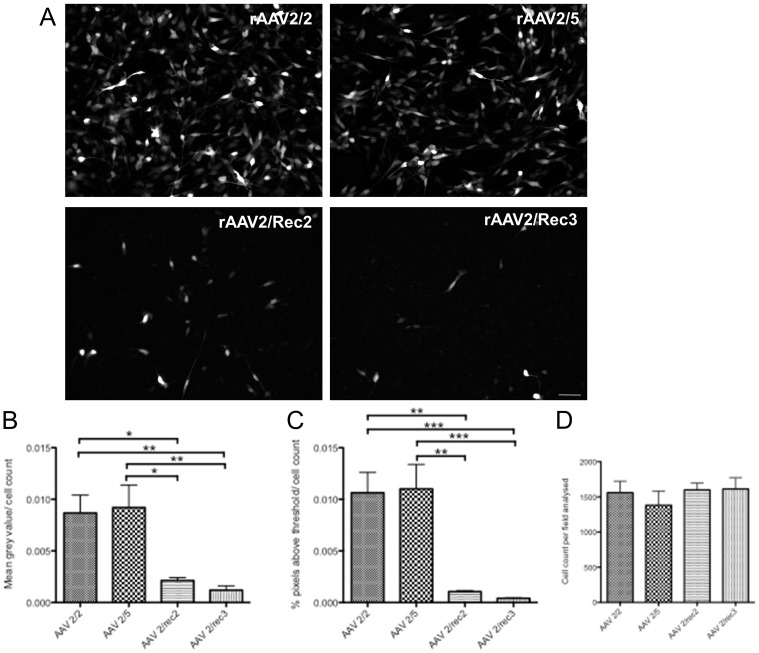
Analysis of Green fluorescent protein (GFP) fluorescence intensity measurement of SH-SY5Y cells *in vitro* 9 days after transfection using different recombinant AAV serotypes. Representative images are shown of GFP fluorescence following transduction with rAAV2/2, rAAV2/5, rAAV2/Rec2 and rAAV2/Rec3 (A). Analysis of grey value (B) and percentage of pixels above threshold (C) are shown in the bottom row, demonstrating significant differences between standard rAAV2/2 and rAAV2/5 serotypes and hybrid recombinant vectors (*p<0.05, **p<0.01, ***p<0.001). In order to ensure levels of cell confluence did not differ between groups and affect transduction, Hoechst-labeled nuclei were counted in each field analyzed, with no significant difference between groups (D). Scale bar 50 µm.

In the human derived neuroblastoma cell line SH-SY5Y, GFP fluorescence intensity as measure for transduction efficiency was significantly different between rAAV serotypes (p = 0.0006; Figure 6B). Grey value analysis showed reduced fluorescence with rAAV2/Rec2 compared to rAAV2/2 (p<0.05) and rAAV2/5 (p<0.05). rAAV2/Rec3 also showed reduced fluorescence compared to rAAV2/2 (p<0.01) and rAAV2/5 (p<0.01). There was no significant difference in pairwise comparisons between rAAV2/2 and rAAV2/5 or between rAAV2/Rec2 and rAAV2/Rec3. Examining pixels above threshold (Figure 6C), rAAV2/Rec2 showed lower transduction efficiency for SH-SY5Y cells than rAAV2/2 (p<0.01) and rAAV2/5 (p<0.01). This was also the case for rAAV2/Rec3 compared to rAAV2/2 and rAAV2/5 (p<0.001 in both). Nuclear count was without significant difference between groups, ensuring similar confluence of the cell layers (Figure 6D).

## Discussion

In this study we have assessed the retinal tropism and transduction efficiency of four recombinant AAV vectors: two standard serotypes (rAAV2/2 and rAAV2/5) and two with novel hybrid recombinant capsids (rAAV2/Rec2 and rAAV2/Rec3). *In vivo* mouse experiments (WT mice and two models of retinal degeneration) were combined with *ex vivo* testing of primate retinal explants and *in vitro* investigation in a human-derived neural cell line (SH-SY5Y). The construction of vectors with hybrid capsids involved recombination of structural domains from vectors of different serotypes previously isolated from primate brain together with AAV8 to create vectors with novel capsids (see methods). We provide data showing that it is possible to interchange viral capsid protein sequences between different AAV serotypes and that this still results in retinal transduction, albeit at a reduced efficiency compared to vectors pseudotyped with wildtype capsids. Although in this pilot study we did not show an increase in transduction efficiency or significant change in tropism with the hybrid recombinant vectors, we propose that this approach in creating hybrid capsids may be a useful tool in generating any number of AAV capsid sequences in order to identify those with the greatest impact on transduction and cellular tropism.

In addition, shuffling specific capsid sequences may be of use in minimizing immune responses to AAV gene therapy. Previous studies have shown that high dose rAAV2/2 administration into the subretinal space in mice leads to production of neutralising antibodies to AAV, which may affect efficacy of treatment to a second eye [13]. In systemic gene therapy using rAAV2 for the treatment of haemophilia B, it has been postulated that the loss of factor IX transgene expression is due to proteasome-mediated cleaving of the AAV capsid, and presentation of capsid peptide sequences via the major histocompatibility complex. This would lead to recognition of transduced cells by CD8+ T cells, and their subsequent destruction [14]. In both cases, the ability to substitute specific capsid antigen sequences, by the technique of capsid shuffling, may be of use in avoiding the immune response to rAAV.

Several variations in AAV genome sequences have been isolated from a variety of body tissues, including the gut, lung and brain [10]. One principal drawback of our study is that the novel capsid sequences (cy5, rh20 and rh39) were identified in primate brain and not in the retina. It was interesting to note that rAAV2/Rec3 which had the highest transduction in neural tissue also showed greatest tropism for retinal ganglion cells in primate retinal explants. This is a similar finding with rAAV2/2 which also has been shown to effectively target brain and retinal ganglion cells [15], [16]. In contrast, rAAV2/8 targets photoreceptors highly efficiently but ganglion cells relatively poorly [17]. In future it might therefore be prudent for retinal gene therapy to attempt isolation by PCR of AAV genome particles directly from primate retina.

AAV2/Rec2 and AAV2/Rec3 were effective in transducing ganglion cells following subretinal vector delivery in the *Pde6b^rd1/rd1^* mouse, and the primate retinal explants. No ganglion cell transduction was seen when these vectors were administered subretinally or intravitreally in WT mice. The former is likely due to the lack of penetration of these vectors to the ganglion cell layer in the presence of a thick ONL, and the latter may be due to an intact inner limiting membrane (ILM), which is known to affect viral transduction. [18] Indeed, the presence of an intact ILM may explain the fact that transduced cells were predominantly observed at the edge of the primate retinal explants, where the ILM would have been disrupted by tissue dissection. rAAV2/Rec2 and rAAV2/Rec3 only weakly transduced photoreceptors. These observations show some of the limitation of *in vitro* experiments. However, the data may provide an effective means before deciding whether to conduct *in vivo* experiments in non-human primates.

Recent work has shown that modification of tyrosine residues can be exploited to significantly enhance AAV transduction in the retina [19]. In our study none of the recombined capsids included serotypes with modification of tyrosine residues, and the number of exposed surface tyrosine residues on rAAV2/Rec2 and Rec3 capsids is comparable to that of rAAV2/8. Hence, it is unlikely that the reduced transduction is due to increased ubiquitin-tagged proteasome-mediated degradation [20], as one might observe if greater numbers of tyrosine residues were present on the hybrid capsid. Both rAAV2/Rec2 and rAAV2/Rec3 vectors use the C terminus of the VP3 region of rAAV2/8 which includes the HI loop, a critical sequence for genome packaging and capsid assembly [21]. Hence this region at least can be excluded as a cause for the reduced transduction seen. It is however possible that impaired synthesis of the viral capsid might result in impaired genome packaging and increase the number of truncated transgenes which might not be detected in the PCR assay used to determine titer. In order to confirm that the hybrid recombinant capsids are able to rearrange into an appropriate AAV structure, it would be necessary to perform more complex analyses of AAV geometry such as X-ray crystallography. However, this technique would only give detail as to the structure of fully matured virions and would be unlikely to highlight issues relating to improper genome packaging or capsid maturation.

None of the four vectors assessed in this study were able to transduce bipolar cells. This is perhaps not a surprising finding because vectors which are highly efficient at transducing photoreceptors, such as rAAV2/5, can achieve high levels of gene expression in the innermost rod photoreceptors but fail to label cells of the adjacent inner nuclear layer [22]. In the *Pde6b^rd1/rd1^* mouse model used here, virtually the entire ONL (i.e. the photoreceptors) had degenerated and hence the failure to target bipolar cells cannot be due to the failure of AAV to access this layer. Ubiquitination of AAV particles may be an important mechanism preventing successful AAV transduction of bipolar cells. This is suggested by the recently achieved effective transduction of bipolar cells using the capsid mutant rAAV2/8 [23] which has one less tyrosine residue at position 503, resulting in reduced ubiquitination compared to the serotypes explored in this study. However, due to the design of our study, such conclusions on the lack of bipolar cell transduction remain speculative.

It was also noted that the transduction of RPE cells and photoreceptors in the *Abca4^−/−^* mouse appears to be significantly lower with all viral serotypes assessed in this study. If this is also true of the human condition then it would also present a relative impediment in using AAV to treat Stargardt disease. Since the *ABCA4* transgene (at 6.7kb) is too large to be packaged into a single AAV vector capsid, one current approach is to use two AAV genomes which can then recombine to form the full-length *ABCA4* transgene after cell entry [24], [25]. In order to achieve this it is likely that much higher levels of transduction will be necessary to compensate for a relatively low rate of transgene recombination. It is not entirely clear why *Abca4^−/−^* mice have reduced transduction with AAV. One explanation might be the different background strain (129S4v/SvJae) compared to the wild type control mice (C57/BL/6). Alternatively it is possible that loss of the transmembrane ABCA4 protein or the increased levels of phosphatidylethanolamine [26] reduce AAV entry into the photoreceptor cell. This explanation may also account for the poor RPE transduction observed, as the number of AAV particles entering RPE cells through phagocytosed outer segment discs would be low if the latter contained low amounts of AAV. It is also possible that the strong accumulation of lipofuscin, which mainly consists of visual cycle byproducts such as N-retinylidene-N-retinyl-ethanolamine (A2E) and other bisretinoids, might somehow impair the transduction efficiency of AAV. Further studies will be required to address this question.

In summary we further explored the concept of pseudotyping viruses to alter retinal cell tropism by mixing capsid proteins. Although the herein tested viruses may not be recommended for further studies due to their lower efficiency compared to established pseudotypes, our methodological approach might be used to identify hybrid rAAV vectors with specific retinal cell tropisms or increased efficiency in certain disease states.

## Methods

### Viral Vectors

Three new recombinant serotypes previously identified, with greater transduction efficiency than rAAV8, were cloned into AAV helper plasmids as previously described [11]. These are termed cy5 (cynomolgus macaque – variant 5), rh20 (rhesus macaque – variant 20) and rh39 and were originally supplied by Guang-Ping Gao and the Gene Therapy Program Vector Core, Department of Medicine, University of Pennsylvania, where further details of the identification of these sequences is available [10].

For the generation of hybrid recombinant capsids, fragments of capsid sequences that matched in all three vectors and AAV8 were shuffled around by using known restriction sites. Particular attention was made to use restriction enzymes that facilitated mixing of the three viral protein (VP) sequences, between the novel primate vectors and AAV8. The N-terminus of the VP1 protein is known to affect AAV infectivity. Hence one of the fragments chosen was derived by HindIII-StuI restriction digest which codes for the first 80 amino acids of VP1 including the phospholipase (PL) A2 site (Figure S2 in File S1). The HindIII-StuI fragments from cy5 were incorporated into Rec1, 3 and 4, whereas the restriction fragments from rh20 or rh39 (which are identical) created a different VP1 for Rec2, 5 and 6. The restriction enzyme MluI cuts nearly in half the coding region for VP2 and VP3 in rh20 and AAV8. SmaI does the same for cy5 and rh20. This allowed the creation of Rec1, 2 and Rec5, 6. Restriction enzyme BamHI cuts off a smaller C-terminal fragment containing the HI loop in rh20, rh39 and AAV8. This allowed the generation of the Rec3 and Rec4 constructs (Figure S1 in File S1).

In a preliminary study the rAAV2/Rec vectors were assayed using standard in vivo protocols comparing transduction efficiency to rAAV2/8. With intravenous infusion, rAAV2/Rec2 showed the greatest transduction of the target tissue (cardiac muscle), and with direct intracerebral administration, rAAV2/Rec3 led to the greatest neuronal cell transduction (unpublished data, Matthew J. During). These vectors (referred to as rAAV2/Rec2 and rAAV2/Rec3) were therefore selected for this study.

To generate hybrid AAV vectors, GFP was cloned into an AAV expression plasmid under the control of the CAG (hybrid CMV-chicken β-actin) promoter and containing woodchuck hepatitis virus post-transcriptional regulatory element (WPRE), and bovine growth hormone (bGH) polyadenylation signal flanked by AAV2 inverted terminal repeats. Human embryonic kidney 293 cells were co-transfected with three plasmids–AAV plasmid, appropriate helper plasmid encoding rep and cap (Rec2 and 3) genes, and adenoviral helper pF Δ6–using standard CaPO4 transfection. rAAV vectors were purified from the cell lysate by ultracentrifugation through an iodixanol density gradient, as previously described [11]. Vectors were titered using real-time PCR (ABI Prism 7700; Applied Biosystems, Foster City, CA) and diluted to 1.0×10^12^ vector genomes (vg)/mL prior to subretinal or intravitreal injection.

rAAV2/2 and rAAV2/5 expressed GFP under control of the same CAG promoter and included WPRE and bovine growth hormone polyadenylation signal as did the hybrid recombinant vectors. The alignment of the rAAV2/Rec2 and rAAV2/Rec3 sequences in comparison to AAV5 and AAV2 is shown in the supporting information (Figure S2 in File S1).

### Mice

Wild type (WT) C57BL/6 mice were provided by the Biomedical Sciences division, University of Oxford. C3H/HeNHsd-*Pde6b^rd1^* (herein referred to as *Pde6b^rd1/rd1^*) mice were purchased from Harlan Laboratories (Hillcrest, UK). Founder *Abca4* knockout mice (129S4/SvJae-*Abca4^tm1Ght^*, herein referred to as *Abca4^−/−^*) were provided by Gabriel Travis, David Geffen School of Medicine, University of California, Los Angeles, USA. [26] and bred locally at the University of Oxford.

Animals were kept in a 12 hour light (<100 lux)/dark cycle, with food and water available *ad libitum*. All procedures were performed under the approval of local and national ethical and legal authorities and in accordance with the Association for Research in Vision and Ophthalmology statements on the care and use of animals in ophthalmic research. The study was approved by the Faculty of Clinical Medicine Ethical Review Committee, and was then assessed, approved and covered by UK Home Office Project licence (number: 30/2808). All procedures were perfomed under general anesthesia, and all efforts were made to minimize suffering.

At the time of intraocular injection, mice were between 6 and 8 weeks old. For surgery and *in vivo* imaging procedures, animals were anesthetized by intraperitoneal injection of 1 mg/kg medetomidine (Dormitor 1 mg/ml, Pfizer, Sandwich, UK) and 60 mg/kg ketamine (Ketaset 100 mg/kg, Fort Dodge, Southampton, UK) and pupils fully dilated with tropicamide 1% eye drops (Bausch & Lomb, Kingston-Upon-Thames, UK) and, for the imaging procedure, phenylephrine eye hydrochloride 2.5% drops (Bausch & Lomb, Kingston-Upon-Thames, UK).

### Intraocular Injections

Intra-ocular injections were performed tangentially through the sclera with a 10 mm 34-gauge needle (Hamilton; Hamilton AG, Bonaduz, Switzerland) mounted on a 5 µl syringe (Hamilton 65 RN, Hamilton AG) under direct visual control using a surgical microscope. A circular cover glass (Ø6 mm, VWR International, Lutterworth, UK) was applied onto the cornea with a carbomer coupling gel (Viscotears, Novartis, Frimley, UK) to ensure good visualization of the fundus. The eye position was controlled and stabilized by holding the superior or inferior rectus muscle with a notched forceps. 1 µl viral vector (1.0×10^12^ vg/mL) was injected in each eye, and complete subretinal or intravitreal delivery was confirmed by direct visualization. After injection, the needle was left in position for an additional 20–30 seconds and then withdrawn quickly to minimize reflux and to allow self-sealing of the scleral tunnel. Each animal received a different viral serotype in each eye, with different syringes and needles used for the different viruses. Between individual injections, the needle was flushed with sterile water.

### Fundus Imaging using a Confocal Scanning Laser Ophthalmoscope

Three weeks after intraocular injection, confocal scanning laser ophthalmoscope (cSLO; Spectralis HRA, Heidelberg Engineering, Heidelberg, Germany) imaging was performed under general anesthesia (as above) according to a modified protocol that has been described in detail previously [27]. In brief, the pupils were fully dilated and a contact lens applied on the cornea. The near-infrared (NIR) reflectance mode (820 nm laser) was used for camera alignment. For recordings from the outer retina, the plane of highest NIR reflectivity or the plane of the nerve fibre layer was identified, respectively. After switching to the autofluorescence mode (excitation wavelength: 488 nm, emission recorded between 500 and 700 nm) and slight focus adjustments (due to the dioptric shift between the different wavelengths), images were recorded with a standardized detector sensitivity of 70 using the automated real time (ART) mode, without image normalization. All images were recorded in the high-resolution mode (1536×1536 pixels) using a 55 degree lens.

### Tissue Collection and Processing

After the imaging procedure, mice were perfusion fixed using 4% paraformaldehyde (PFA, Thermo Fisher, Loughborough, UK) in PBS. After enucleation, the cornea and lens were removed under direct visualisation with an operating microscope in 4% PFA in PBS. After fixation overnight, the eyecups were cryoprotected using a 10–30% sucrose gradient. Eyecups were embedded in optimal cutting temperature (OCT) compound (Tissue-Tek, Sakura Finetek, The Netherlands), frozen on dry ice and stored at −80°C until sectioning. Eyecups were cryosectioned into 16 µm sections and affixed to poly-L-lysine coated glass slides (Polysine®; Thermo Scientific, Loughborough, UK). The sections were air-dried and then stored at −20°C until further histological processing.

### Retinal Explant Culture

Eyes were enucleated from a 12-year-old rhesus macaque (*Macaca mulatta*) immediately *post-mortem* and transferred into room temperature complete culture media consisting of Neurobasal A, L-glutamine (0.08 mM), penicillin (100 U/mL), streptomycin (100 U/mL) and serum free supplements B27 (2%) and N2 (1%, all Invitrogen Ltd, Paisley, UK). The anterior segment and lens were removed and the retina detached from the RPE. Subsequently, the retina was divided into 2–3 mm segments and transferred into an organotypic system for long term culture, as described previously [12], [28] One day post explantation (D1), 20 µl rAAV2/Rec2 (1.0×10^12^ vg/mL) and rAAV2/Rec3 (1.0×10^12^ vg/mL) vectors were applied to the retinal explants during media change. Media (700 µL) was refreshed every third day and GFP expression evaluated using an inverted epi-fluorescence microscope (Leica DM IL, Leica, Wetzlar, Germany). Images of live explants were acquired at D10 after removal of media to ensure that explants remained flat on the tissue culture membranes and that background autofluorescence due to the presence of media was minimal. Following imaging, explants were removed from tissue culture and fixed for 40 min in 4% PFA followed by incubation overnight at 4°C in 30% sucrose. Explants were subsequently embedded in OCT, frozen on dry ice and stored at −80°C until sectioning.

### Histology and Immunohistochemistry

After hydration and 3×5 min washes in 0.01 M PBS, retinal sections were blocked for 1 hour at room temperature in PBS +0.1% Triton X-100+10% donkey serum. After 3×5 min washes, the sections were incubated at 4°C overnight with the primary antibody and subsequently for 2 hours at room temperature with the species–appropriate secondary antibody (both in PBS +0.1% Triton X-100+1% serum). After each step, sections were rinsed for 2×5 min in PBS +0.05% Tween20, followed by 1×5 min in PBS alone. All sections were counter-stained with Hoechst 33342 (Invitrogen) 1∶5000 and mounted with an antifade reagent (Prolong Gold; Invitrogen). All antibodies used for immunohistochemistry are specified in table 2. Alexa Fluor 555 donkey anti-rabbit IgG and Alexa Fluor 568 donkey anti-goat IgG (Invitrogen) were used as secondary antibodies as appropriate.

**Table 2:** Details of primary antibodies used for immunohistochemistry.

Staining	Antigen	Host	Source (product code)	Dilution
Cone arrestin	Synthetic linear peptide	Rabbit	Millipore (AB15282)	1∶1000
Calbindin	28kDa calbindin-D protein purified from rat kidney	Rabbit	Abcam (ab11426)	1∶1000
GFAP	GFAP isolated from cow spinal cord	Rabbit	Abcam (ab7779)	1∶1000
PKCα	C-terminus of human PKCα	Rabbit	Epitomics (1510-1)	1∶1000
Brn-3a	N-terminus of human Brn-3a	Goat	Santa Cruz (sc-31984)	1∶250

Milipore, Billerica, MA. Abcam, Cambridge, UK. Epitomics, Burlingame, CA. Santa Cruz Biotechnology, Santa Cruz, CA.

### Confocal Microscopy

Retinal sections were viewed on a confocal microscope (LSM710; Zeiss, Jena, Germany). GFP-positive cells were located using epifluorescence illumination before taking a series of overlapping XY optical sections, of approximately 0.5 µm thickness. The fluorescence of Hoechst, GFP and Alexa-555 or 568 were sequentially excited using 350 nm UV laser, 488 nm argon laser and the 543 nm HeNe laser, as appropriate. A stack was built to give an XY projection image as appropriate. Image processing was performed using Volocity (Perkin Elmer, Cambridge, UK) and Image J (Version 1.43, National Institute of Health, http://rsb.info.nih.gov/ij).

### Light Microscopy

For the purpose of quantitative analysis of GFP expression, images were taken using the Leica DM IL inverted epifluorescence microscope. Images were obtained at x20 magnification using identical acquisition settings including exposure time, and were saved at a resolution of 1200×1600 pixels.

### Image Analysis

Image analysis was performed on 8-bit images using ImageJ. Grey levels on cSLO images were measured within a circle of 300 pixel diameter. This area was placed within the region of vector delivery, avoiding the needle entry site where associated retinal damage often led to localized increased transduction.

Grey levels on histological fluorescence images were analyzed separately for the RPE, the photoreceptor layer, and the inner retina (including the outer plexiform layer and the inner nuclear layer). This allowed a quantitative analysis of GFP expression within individual retinal layers and thus to assess vector tropism. Retinal pigment epithelium (RPE) cells were always most efficiently transduced. Thus to ensure images were not over-exposed, the exposure time for images analyzed for GFP-fluorescence in the RPE was one quarter of that used for assessing transduction of neuronal cells. Regions of defined sizes (RPE: 20×200 pixels, outer nuclear layer: 50×250 pixels, inner retina: 100×250 pixels) were analyzed within areas of highest GFP expression, and the mean grey level was calculated from a plot profile. In addition, the number of pixels above threshold was calculated from histogram analysis. The threshold was set conservatively using areas outside the injection site as reference.

### Cell Culture and in vitro Transduction

SH-SY5Y cells (obtained from the American tissue culture collection) were cultured in complete RPMI-1640 media containing L-glutamine (2 nM), penicillin (100 units/mL), streptomycin (100 µg/mL) all from Sigma-Aldrich UK, and 10% fetal calf serum (GIBCO, Invitrogen, UK). Cells were maintained at 37°C in a 5% CO2 environment.

Cells were seeded in 96 well culture dishes at a density of 5×10^4^ cells per well. One day after plating, media was changed to that containing 1.6×10^−8^ M Tetradecanoylphorbol-13-acetate (TPA) and 10^−5^ M retinoic acid (RA) to induce neuron-specific differentiation and morphological changes in the SH-SY5Y cells as previously shown [29].

After 48 hours, media was changed and 1 µL of virus (1.0×10^12^ vg/mL ) was added per well, giving an overall multiplicity of infection (MOI) of approximately 2×10^4^ vg/cell. The media was changed every 48 hours and RA and TPA were maintained in the media throughout.

Cells were imaged daily to detect GFP expression using an inverted epifluorescence microscope (Leica DM IL). Images were obtained at x20 magnification using identical acquisition settings including exposure time, and were saved at a resolution of 1200×1600 pixels. 9 days after transfection, cells were fixed using 4% paraformaldehyde in PBS and Hoechst 33342 was used for nuclear staining.

Post-fixation images were taken as above, and 8-bit images were analyzed using Image J software. The mean grey level of each image was calculated from a plot profile and the number of pixels above threshold calculated from histogram analysis. Control values (from images taken of wells where no virus was added) were subtracted from the data points to minimize background and also were used to set the threshold. The number of Hoechst-labelled cell nuclei in each field analysed were counted, and grey level and pixel above threshold values were adjusted accordingly, to ensure that any difference in cell number was accounted for.

### Statistical Analysis

Mean grey levels on cSLO and histological fluorescence images were compared using two-way ANOVA using strain and rAAV serotype as factors. Mean GFP fluorescence intensity in SH-SY5Y cells in vitro was compared using one-way ANOVA. The Bonferroni post hoc test was applied in all instances, and the significance level was set at 0.05.

## Supporting Information

File S1 **Supporting Information.**
(DOC)
