# Supplementary material for: Assessment of Tropism and Effectiveness of New Primate-Derived Hybrid Recombinant AAV Serotypes in the Mouse and Primate Retina
Source: PLoS One. 2013 Apr 9;8(4):e60361. doi: 10.1371/journal.pone.0060361 (PMC3621895; doi:10.1371/journal.pone.0060361)
Supplement: File S1 — Supporting Information. (DOC) [file pone.0060361.s001.doc]

**Assessment of tropism and effectiveness of new primate derived hybrid recombinant AAV serotypes in the mouse and primate retina**

**- Supporting information -**

**Figure S1**

**Recombinant AAV capsid (Rec) serotypes**

Structure of the six recombinant capsid (Rec) serotypes initially screened showing regions of overlap and restriction sites. The position of the wildtype viral protein (VP) 1-3 sequences are shown in black. The C-terminal half of the VP1 and VP2 proteins includes both the area that corresponds to the heparin binding domain in AAV2 and the HI loop. Whereas, the N-terminal half contains the nuclear localization signal like sequence, as well as several other highly variable domains. Rec2 and Rec3 showed greatest transduction efficacy in cardiac muscle and brain respectively and were selected for this study in the retina.

**Figure S2**

**Rec2/ Rec3/ AAV2/ AAV5 VP protein alignment**

Rec2 **M**AADGYLPDWLEDNLSEGIREWWDLKPGAPKPKANQQKQDDGRGLVLPGYKYLGPFNGLD 60

Rec3 **M**AADGYLPDWLEGNLSEGIREWWDLKPGAPKPKANQQKQDDGRGLVLPGYRYLGPFNGLD 60

AAV2 **M**AADGYLPDWLEDTLSEGIRQWWKLKPGPPPPKPAERHKDDSRGLVLPGYKYLGPFNGLD 60

AAV5 **M**SFVDHPPDWLE-EVGEGLREFLGLEAGPPKPKPNQQHQDQARGLVLPGYNYLGPGNGLD 59

VP1

*StuI*

Rec2 KGEPVNAADAAALE**HDKAY**DQQLKAGDNPYLRYNHADAEFQERLQED**T**SFGGNLGRAVFQ 120

Rec3 KGEPVN**E**ADAAALE**HDKAY**DQQLKAGDNPYLRYNHADAEFQERLQED**T**SFGGNLGRAVFQ 120

AAV2 KGEPVN**E**ADAAALE**HDKAY**DRQLDSGDNPYLKYNHADAEFQERLKED**T**SFGGNLGRAVFQ 120

AAV5 RGEPVNRADEVARE**HDISY**NEQLEAGDNPYLKYNHADAEFQEKLADD**T**SFGGNLGKAVFQ 119

PLA2 VP2

Rec2 AKKRVLEPLGLVEEGAKTAPGKKRPVEPSPQRSPDSSTGIGKTGQQ**PAKKRLN**FGQTGDS 180

Rec3 AKKRVLEPLGLVEEAAKTAPGKKRPVEPSPQRSPDSSTGIGKKGQQ**PAKKRLN**FGQTGDS 180

AAV2 AKKRVLEPLGLVEEPVKTAPGKKRPVEHSPV-EPDSSSGTGKAGQQ**PARKRLN**FGQTGDA 179

AAV5 AKKRVLEPFGLVEEGAKTAPTGKRIDDHFPKRKKART----EEDSKPS-------TSSDA 168

NLS

Rec2 ESVPDPQPIGEPPAGP-SGLGSGT**M**AAGGGAPMADNNEGADGVGSSSGNWHCDSTWLGDRV 240

Rec3 ESVPDPQPIGEPPAGP-SGLGSGT**M**AAGGGAPMADNNEGADGVGSSSGNWHCDSTWLGDRV 240

AAV2 DSVPDPQPLGQPPAAP-SGLGTNT**M**ATGSGAPMADNNEGADGVGNSSGNWHCDSTWMGDRV 239

AAV5 EAGPSGSQQLQIPAQPASSLGADT**M**SAGGGGPLGDNNQGADGVGNASGDWHCDSTWMGDRV 229

VP3

Rec2 ITTSTRTWALPTYNNHLYKQISNGTSGGSTNDNTYFGYSTPWGYFDFNRFHCHFSPRDWQ 300

Rec3 ITTSTRTWALPTYNNHLYKQISNGTSGGSTNDNTYFGYSTPWGYFDFNRFHCHFSPRDWQ 300

AAV2 ITTSTRTWALPTYNNHLYKQISSQ-S-GASNDNHYFGYSTPWGYFDFNRFHCHFSPRDWQ 297

AAV5 VTKSTRTWVLPSYNNHQYREIKSGSVDGS-NANAYFGYSTPWGYFDFNRFHSHWSPRDWQ 288

Rec2 RLINNNWGFRPKRLNFKLFNIQVKEVTQNEGTKTIANNLTSTIQVFTDSEYQLPYVLGSA 360

Rec3 RLINNNWGFRPKRLSFKLFNIQVKEVTQNEGTKTIANNLTSTIQVFTDSEYQLPYVLGSA 360

AAV2 RLINNNWGFRPKRLNFKLFNIQVKEVTQNDGTTTIANNLTSTVQVFTDSEYQLPYVLGSA 357

AAV5 RLINNYWGFRPRSLRVKIFNIQVKEVTVQDSTTTIANNLTSTVQVFTDDDYQLPYVVGNG 348

Rec2 HQGCLPPFPADVFMIPQYGYLTLN--NGSQAVGRSSFYCLEYFPSQMLRTGNNFEFSYQFED 420

Rec3 HQGCLPPFPADVFMIPQYGYLTLN--NGSQAVGRSSFYCLEYFPSQMLRTGNNFEFSYTFED 420

AAV2 HQGCLPPFPADVFMVPQYGYLTLN--NGSQAVGRSSFYCLEYFPSQMLRTGNNFTFSYTFED 417

AAV5 TEGCLPAFPPQVFTLPQYGYATLNRDNTENPTERSSFFCLEYFPSKMLRTGNNFEFTYNFEE 410

Rec2 VPFHSSYAHSQSLDRLMNPLIDQYLYYLSRTQSTGGTAGTQQLLFSQAGPNNMSAQAKNW 480

Rec3 VPFHSSYAHSQSLDRLMNPLIDQYLYYLSRTQSTGGTQGTQQLLFSQAGPANMSAQAKNW 480

AAV2 VPFHSSYAHSQSLDRLMNPLIDQYLYYLSRTNTPSGTTTQSRLQFSQAGASDIRDQSRNW 477

AAV5 VPFHSSFAPSQNLFKLANPLVDQYLYRFVSTNNTGG------VQFNKNLAGRYANTYKNW 464

*MluI*

Rec2 LPGPCYRQQRVSTTTGQNNNSNFAWTAGTKYHLNGRNSLANPGIAMATHKDDEERFFPSN 540

Rec3 LPGPCYRQQRVSTTLSQNNNSNFAWTGATKYHLNGRDSLVNPGVAMATHKDDEERFFPSS 540

AAV2 LPGPCY**R**QQ**R**VSKTSADNNNSEYSWTGATKYHLNGRDSLVNPGPAMASHKDDEE**K**FFPQS 537

AAV5 FPGPMGRTQGWNLGSGVNRASVSAFATTNRMELEGASYQVPPQPNGMTNNLQGSNTYALE 524

Rec2 GILIFGKQNA-ARDNADY-SDVML-TSEEEIKTTNPVATEEYGIVADNLQQQNTAPQIGTVNS 600

Rec3 GVLMFGKQGA-GRDNVDY-SSVML-TSEEEIKTTNPVATEQYGVVADNLQQTNTGPIVGNVNS 600

AAV2 GVLIFGKQGS-EKTNVDI-EKVMI-TDEEEIRTTNPVATEQYGSVSTNLQ**R**GN**R**QAATADVNT 597

AAV5 NTMIFNSQPANPGTTATYLEGNMLITSESETQPVNRVAYNVGGQMATNNQSSTTAPATGTYNL 587

**R** 484, 487, 585, 588 and **K** 532 – heparin binding domain

*BamHI*

Rec2 QGALPGMVWQNRDVYLQGPIWAKIPHTDGNFHPSPLMGGFGLKHPPPQILIKNTPVP**ADP** 660

Rec3 QGALPGMVWQNRDVYLQGPIWAKIPHTDGNFHPSPLMGGFGLKHPPPQILIKNTPVP**ADP** 660

AAV2 QGVLPGMVWQDRDVYLQGPIWAKIPHTDGHFHPSPLMGGFGLKHPPPQILIKNTPVP**ANP** 657

AAV5 QEIVPGSVWMERDVYLQGPIWAKIPETGAHFHPSPAMGGFGLKHPPPMMLIKNTPVP**GN-** 647

Rec2 **PTTFNQSKLN**SFITQYSTGQVSVEIEWELQKENSKRWNPEIQYTSNYYKSTSVDFAVNTE 720

Rec3 **PTTFNQSKLN**SFITQYSTGQVSVEIEWELQKENSKRWNPEIQYTSNYYKSTSVDFAVNTE 720

AAV2 **STTFSAAKFA**SFITQYSTGQVSVEIEWELQKENSKRWNPEIQYTSNYYKSTSVDFAVNTE 717

AAV5 **ITSFSDVPVS**SFITQYSTGQVTVEMEWELKKENSKRWNPEIQYTNNYNDPQFVDFAPDST 706

HI loop

Rec2 GVYSEPRPIGTRYLTRNL 738

Rec3 GVYSEPRPIGTRYLTRNL 738

AAV2 GVYSEPRPIGTRYLTRNL 735

AAV5 GEYRTTRPIGTRYLTRPL 724

Critical residues are in **bold**: PLA2 – phospholipase A2 site (endosomal release); NLS – nuclear localization signal; HI Loop – protein sequence connecting the H and I beta-strands of the VP3 subunit (genome packaging and assembly). Restriction sites are shown above sequences. Changes between the sequences are highlighted in red. Note that most of the sequence variation of Rec2 and Rec3 is with AAV5, whereas they are not as dissimilar with AAV8.
